# Sugar Reformulation in Solid Foods: Limitations and Challenges

**DOI:** 10.1111/nbu.70004

**Published:** 2025-04-11

**Authors:** Jimmy Chun Yu Louie

**Affiliations:** ^1^ Department of Allied Health, School of Health Sciences Swinburne University of Technology Hawthorn Victoria Australia

**Keywords:** food policy, free sugar, obesity prevention, solid foods, sugar reformulation, sugar‐sweetened beverages

## Abstract

The global obesity epidemic remains a significant public health challenge, prompting various interventions to address its complexity. Among these, sugar reformulation in foods has gained traction as a potential strategy. While successful in sugar‐sweetened beverages (SSBs), applying this approach to solid foods presents unique challenges and potential drawbacks. This article critically discusses the efficacy of sugar reformulation in solid foods as an obesity prevention strategy. The discussion explores the differential impacts of sugars from SSBs versus solid foods, technical constraints in reformulation and possible unintended outcomes. Additionally, the article evaluates the limitations of modelling studies that advocate for sugar reformulation, emphasising the importance of a balanced and evidence‐based perspective on its role in addressing obesity.

## 1 Introduction

The global obesity epidemic continues to be one of the most pressing public health challenges of our time, affecting millions of individuals across all age groups and socioeconomic strata (Koliaki, Dalamaga, and Liatis 2023). As policymakers and health professionals grapple with this complex issue, various interventions have been proposed and implemented to curb the rising tide of obesity (Bagnall et al. 2019; Temple 2023).

Public health organisations, policymakers and researchers widely support sugar reformulation (i.e., reducing the sugar content of processed foods) as a key strategy to reduce sugar intake and address obesity (Basto‐Abreu et al. 2018; Eyles et al. 2020; Moynihan and Miller 2020; Pepper, Hart, and Hodgkins 2023; World Health Organization 2022). This approach aligns with the World Health Organization's recommendation to reduce free sugars intake to less than 10% of total energy intake, with a further reduction to below 5% suggested for additional health benefits (World Health Organization 2015). Advocates suggest that reducing sugar in foods can help lower energy intake, aiding in weight management and obesity prevention (Stanner and Spiro 2020). This approach has proven effective with sugar‐sweetened beverages (SSBs), where reformulation has led to significant reductions in sugar consumption (Allais et al. 2023; Bandy et al. 2020) and in obesity prevalence in some groups of children (Rogers et al. 2023). However, extending this strategy to solid foods poses distinct challenges and risks that need closer examination (Goldfein and Slavin 2015). These challenges raise questions about whether sugar reduction in solid foods can achieve similar levels of success as seen with SSBs.

This article contends that sugar reformulation in processed solid foods, while a theoretically promising intervention, is not a sensible solution for obesity prevention and, in some cases, may be less effective than anticipated in reducing energy intake. While sugar reduction is not without merit, this article challenges the assumption that reformulation will significantly lower population energy intake, as some modelling studies suggest. The reality is that reformulating solid foods is much more complex than reformulating beverages, often becoming a slogan that is easier said than done. This article does not suggest that free sugar is harmless but questions whether reformulation is as impactful or cost effective as often assumed by policymakers, public health advocates and some researchers. The minority view previously expressed (Cooper 2017; Cooper 2012), and further discussed here, raises legitimate concerns about whether sugar reformulation can meaningfully reduce energy consumption in real‐world settings. These concerns are not universally accepted, and critics sometimes dismiss them as undermining public health efforts, particularly when they originate from sources perceived to have industry affiliations (Schillinger and Kearns 2017; Stanhope 2022). Such dismissals shut down valuable debate about the unintended consequences of well‐meaning interventions. This article seeks to spark a critical conversation among food scientists and public health experts about the challenges of sugar reformulation in solid foods and its place in obesity prevention. It emphasises the need for rigorous scientific investigation and open dialogue in shaping effective public health strategies.

## 2 Differential Impact of Sugars From Sugar‐Sweetened Beverages and Solid Foods

The rationale behind sugar reduction as a strategy to combat obesity stems from the assumption that excessive sugar consumption, which provides ‘empty calories’, may lead to weight gain (Faruque et al. 2019). However, the relationship between sugar intake and obesity is far more intricate than the simplified narratives often presented in public health campaigns and media discussions (Rippe and Angelopoulos 2016), especially when considering sugars from solid foods versus SSBs (Table 1) (Yan, Chan, and Louie 2022b). Evidence thus far does not support the notion that sugars from SSBs and solid foods are equally harmful (Yan, Chan, and Louie 2022a; Yan, Chan, and Louie 2022b). Consumption of SSBs has been more strongly associated with weight gain and obesity than sugars from solid foods (Khan and Sievenpiper 2016; Yan, Chan, and Louie 2022b). Individuals are more likely to compensate for energy consumed from solid foods in subsequent meals (Flynn et al. 2022), whereas liquid calories from SSBs often lead to overconsumption (Malik and Hu 2022). This distinction highlights the importance of tailoring sugar reduction strategies to specific food categories rather than assuming uniform effectiveness across all types of foods (Erickson and Slavin 2015a; Yan and Louie 2023).

**TABLE 1:** Comparison of the impact of sugars from sugar‐sweetened beverages (SSBs) versus solid foods on various metabolic and behavioural factors.

Factor	SSBs	Solid foods containing sugar
*Satiety*	Low satiety effect; does not trigger fullness signals effectively	Higher satiety due to fibre content and need for mastication
*Caloric compensation*	Poor compensation; calories from SSBs often add to total intake	Better compensation; calories more likely to be offset in subsequent meals
*Consumption speed*	Quickly consumed, leading to rapid sugar absorption	Slower consumption due to chewing, allowing for gradual sugar release
*Portion control*	Easy to overconsume; less visual cues for portion size	Easier to control portions due to physical volume
*Metabolic impact*	Strongly associated with insulin resistance and metabolic syndrome	Less strongly associated with metabolic disturbances when consumed as part of a balanced diet
*Association with weight gain*	Strong association with obesity and weight gain	Weaker association with obesity when consumed in moderation

## 3 Sugar Reformulation—Current Status Around the World

Sugar reformulation targets have become a global trend as countries aim to combat rising obesity rates and improve public health (Table 2). While many initiatives initially focused on SSBs, it is now commonly believed that a more comprehensive approach is necessary to address excessive free sugar consumption across a wide range of food categories.

**TABLE 2:** Voluntary sugar reformulation targets of various countries.

Country	Types of foods included	Target		
United Kingdom (Public Health England 2015)	– Breakfast cereals – Yogurts – Biscuits – Cakes – Morning goods (e.g., croissants, English muffins, waffles) – Puddings – Ice cream, lollies and sorbets – Chocolate confectionery – Sweet confectionery – Sweet spreads and sauces (e.g., chocolate spread, peanut butter, dessert toppings)	Reduction by 20% for all included food groups		
The Netherlands (National Institute for Public Health and the Environment)		**Low (g/100 g)**	**Moderate (g/100 g)**	**Max (g/100 g)**
	– Luxury bread, natural and sweet	16	26	30
	– Luxury bread and savoury	1.7	2.1	2.9
	– Breakfast cereals	10.5	15.5	20.5
	– Dairy drinks, yogurt, quark and custard	8.5	11	12.5
	– Pudding, mousse and desserts	15	19.5	21.5
	– Cakes	28	33	38.5
	– Cookies	28	33	38.5
	– Breakfast cake	35.5	39	42
	– Grain, muesli, fruit and energy bars	20	25	32
	– Cake and pastry	17.5	22	28
	– Chocolate	46	52	56
	– Candy	54	64	74
	– Sorbet ice cream	22	24	27
	– Ice cream and dairy/plant‐based	22	24	27
	– Sweet sauces	52	58	64
	– Soft, sports and energy drinks	4.5	6.5	9
	– Chocolate spreads	41.5	55	61
	– Nut spreads	8	9.5	12
Australia (Coyle et al. 2021)	– Breakfast cereals with fruit	22.5 g/100 g and > 20% reduction if over 28 g/100 g		
	– Breakfast cereals without fruit	20 g/100 g and > 20% reduction if over 25 g/100 g		
	– Flavoured dairy milk	9 g/100 mL		
	– Flavoured dairy alternatives	5 g/100 mL		
	– Muesli and snack bars	25 g/100 g and > 15% reduction if over 28.5 g/100 g		
	– Flavoured water, mineral water and iced tea	5 g/100 mL		
	– Carbonated soft drinks and energy drinks	10% reduction if > 10 g/100 mL		
	– Fruit drinks	9.5 g/100 mL		
	– Sweetened dairy yogurt	12.5 g/100 g		
New Zealand (Heart Foundation (New Zealand) 2024)	– Breakfast cereals (with fruit)	22.5 g/100 g		
	– Breakfast cereals (without fruit)	20 g/100 g		
	– Savoury snacks (popcorn)	25 g/100 g		
	– Cooking sauces (Asian style, excluding sweet types)	14 g/serve		
	– Cooking sauces (Asian style, including sweet types)	20 g/serve		
	– Cooking sauces (tomato‐based pasta sauces)	6 g/100 g		
	– Cooking sauces (non‐tomato–based sauces)	3 g/100 g		
	– Table sauce (tomato sauce/ketchup, canned/glass packaging)	20 g/100 g		
	– Table sauce (tomato sauce/ketchup and plastic packaging)	23 g/100 g		
	– Beans in sauce (baked beans)	5 g/100 g		
	– Beans in sauce (other beans in sauce)	5 g/100 g		
	– Canned spaghetti	4.5 g/100 g		
	– Cereal and nut/seed bars	25 g/100 g		
	– Sweetened dairy‐based yogurt & dairy food	8.5 g/100 g		
	– Sweetened plant‐based yogurt	5 g/100 g		
	– Flavoured dairy milk	7.0 g/100 mL		
	– Flavoured plant‐based beverages	5 g/100 mL		
US (New York City) (Department of Health (New York City) 2021)	– Soft drinks	5.4 g sugar per 100 mL		
	– Sweetened milk	4.8 g sugar per 100 mL		
	– Sweetened milk substitutes	2.9 g sugar per 100 mL		
	– Breakfast pastries	21.7 g sugar per 100 g		
	– Cakes	31.9 g sugar per 100 g		
	– Cookies	28.5 g sugar per 100 g		
	– Dry mixes	39.6 g sugar per 100 g		
	– Granola bars	21.7 g sugar per 100 g		
	– Refrigerated and frozen desserts	16.6 g sugar per 100 g		
	– Sweet candies	47.4 g sugar per 100 g		
	– Chocolate candies	42.9 g sugar per 100 g		
	– Breakfast cereals	22.0 g sugar per 100 g		
	– Condiments	17.5 g sugar per 100 g		
	– Dessert syrups and toppings	43.4 g sugar per 100 g		
	– Yoghurts	5.2 g sugar per 100 g		
Belgium (Kleis, Schulte, and Buyken 2020)	– Soft drinks	10% reduction		
	– Dairy products	8% reduction		
	– Breakfast cereals	4% reduction		
Switzerland (Kleis, Schulte, and Buyken 2020)	– Yogurts	10% reduction		
	– Breakfast cereals	15% reduction		
Spain (Spanish Ministry of Health 2020)	– Dairy products	10% reduction		
	– Meat products	10% reduction		
	– Fruit nectars	10% reduction		
	– Sauces	10% reduction		
	– Soft drinks	10% reduction		
	– Chocolate‐flavoured children's breakfast	10% reduction		

	– Indulgence dairy products	3.5% reduction		
	– Mayonnaise	18% reduction		
	– Cakes and pastries	5% reduction		
	– Biscuits	5% reduction		
	– Water‐based children's ice‐cream	5% reduction		
	– Pre‐packed bread	5% reduction		
– Sauces	5% reduction		
France, Italy. Lithuania. Luxembourg, Malta and Norway (Kleis, Schulte, and Buyken 2020)	– Products of participating companies	Different fixed percentages to reduce the free sugars in Different product groups		

The United Kingdom has set one of the most ambitious and wide‐ranging sugar reduction targets. Their voluntary programme aims for a 20% reduction in sugar content across multiple food categories, including SSBs, yogurts, breakfast cereals, biscuits, cakes, puddings, ice creams and confectionery (Public Health England 2015). This broad approach acknowledges that sugar intake comes from diverse sources in the diet. However, reports from Public Health England on the effectiveness of the Sugar Reduction Programme revealed that reductions in sugar content in some product categories were offset by increased consumption of other categories, resulting in only a modest overall reduction in sugar intake (Office for Health Improvement and Disparities (UK) 2022). This finding highlights the difficulty of achieving dietary change by stealth through reformulation alone, emphasising the need for complementary strategies to guide consumer behaviour.

Similarly, other countries have adopted multicategory approaches to sugar reduction. The Netherlands has established specific sugar content limits for various food categories, including bread, breakfast cereals, dairy products, desserts and confectionery (National Institute for Public Health and the Environment 2024). Australia has set reduction targets for breakfast cereals, dairy products and beverages, with some categories having percentage‐based reductions and others having specific gram‐per‐100 g limits (Coyle et al. 2021). New Zealand (Heart Foundation (New Zealand) 2024) has implemented similar targets across a wide range of food categories, while the United States (specifically New York City) has set sugar content limits for various products, including soft drinks, sweetened milk, baked goods and confectionery (Department of Health 2021). European countries such as Belgium (Kleis, Schulte, and Buyken 2020), Switzerland (Kleis, Schulte, and Buyken 2020) and Spain (Spanish Ministry of Health 2020) have focused on percentage‐based reductions in sugar content for specific food categories, with targets ranging from 3.5% to 18% depending on the product type. France, Italy, Lithuania, Luxembourg, Malta and Norway have adopted a flexible approach, allowing participating companies to set their own reduction percentages for different product groups (Kleis, Schulte, and Buyken 2020). These broader targets pose significant technological hurdles for food manufacturers, discussed in the next section.

## 4 Technical Challenges and Paradoxical Effects

Public health discussions about sugar reduction tend to focus primarily on nutrition science, overlooking the food science aspects of sugar. Nutrition‐based strategies often fail to consider the technical challenges of reformulating foods (Goldfein and Slavin 2015; Russell et al. 2022). This disconnect between nutrition and food science can result in recommendations that, while well intentioned, are impractical or even counterproductive (Yan and Louie 2023).

**FIGURE 1:**
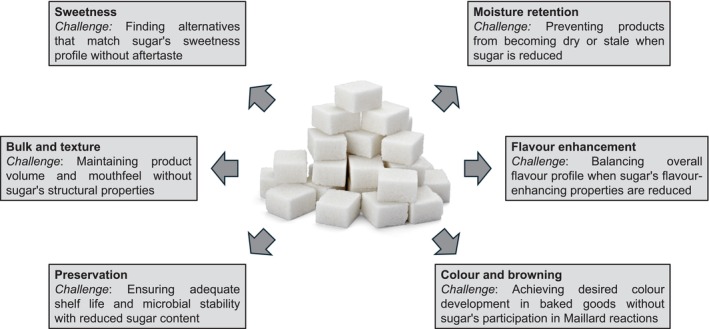
Functions of sugar in food products and the challenges of replacing each function.

Reformulating processed solid foods to reduce sugar content presents numerous technical challenges that underscore the complexity of altering processed food composition (Table 3). Sugar plays multifaceted roles in food products, contributing to sweetness, texture, mouthfeel, preservation and palatability, and its reduction often necessitates using alternative ingredients or processing methods (Figure 1) (Cooper 2017; Erickson and Carr 2020; Goldfein and Slavin 2015; McKenzie and Lee 2022; Sahin et al. 2019; Yan and Louie 2023), leading to several issues with potentially unintended consequences.

**TABLE 3:** Considerations for sugar reduction in sugar‐sweetened beverages (SSBs) versus solid foods.

Considerations	SSBs	Solid foods containing sugar
*Use of non‐nutritive sweeteners*	Highly effective; minimal impact on taste and texture	Less effective; may alter taste, texture and mouthfeel significantly
*Gradual sugar reduction*	Moderately effective; consumers adapt to less sweet taste over time	Less effective; may significantly impact product quality and consumer acceptance
*Functional replacement*	Not typically necessary; sweetness is primary function	Highly complex; must address multiple functional roles of sugar
*Impact on calorie content*	Direct reduction in calories	May lead to minimal change or even increase in calories due to compensatory ingredients
*Consumer acceptance*	Generally high; minimal change in sensory properties	Variable; often lower due to noticeable changes in product characteristics
*Product stability and shelf life*	Minimal impact	Significant challenges; may require additional preservatives
*Cost of reformulation*	Relatively low	Often high due to need for multiple‐ingredient substitutions
*Regulatory compliance*	Straightforward; mainly focused on sweetener regulations	Complex; must consider multiple ingredient changes and labelling requirements
*Health halo effect*	Moderate risk; consumers may overconsume ‘diet’ beverages	High risk; reduced‐sugar solid foods are often perceived as healthier regardless of overall nutritional profile
*Impact on satiety*	Minimal change; SSBs generally provide low satiety	May decrease satiety, potentially leading to overconsumption
*Effectiveness in reducing overall sugar intake*	High; direct reduction in sugar consumption	Moderate to low; may lead to compensatory behaviours or increased consumption of other sugar sources

First and foremost, reduced‐sugar products may be less appealing to consumers (Erickson and Carr 2020), potentially driving them towards other calorie‐dense options or leading to compensatory behaviours that negate any potential benefits of sugar reduction (Cengiz and Rojas 2024). Markey, Lovegrove, and Methven (2015) found that reducing sugar content in baked goods significantly decreased consumer acceptability, highlighting the challenge of balancing health goals with consumer preferences. Moreover, efforts to maintain product consistency and appeal after sugar reduction can lead to increased fat content, particularly in baked goods, confectionery and dairy products (Mahato et al. 2020; McKenzie and Lee 2022; Sahin et al. 2019). For example, Biguzzi, Lange, and Schlich (2015) found that fat content often needed to be increased to maintain sensory properties in sugar‐reduced cookies. This substitution can result in products that, while lower in sugar, are higher in overall energy density (Cooper 2017; Cooper 2012), negating the energy reduction from sugar removal.

The role of sugar as a natural preservative in many products adds another layer of complexity. Reducing sugar content can compromise these protective effects, necessitating additional preservatives or alternative processing methods (Goldfein and Slavin 2015). This adds complexity to the manufacturing process and may conflict with growing consumer preferences for ‘clean label’ products with fewer additives (Asioli et al. 2017). Furthermore, reformulation often requires significant investment in research and development and changes to manufacturing processes (Onyeaka et al. 2023). These high costs may be prohibitive for smaller manufacturers and could lead to higher prices for consumers (Cooper 2017). Since obesity disproportionately affects lower‐income groups (Kumanyika 2019), price increases on reformulated products could undermine efforts to make healthier options accessible, making the strategy counterproductive.

Sugar taxes, primarily targeting sugary drinks, have led to sugar reductions in some regions, but have not consistently lowered population obesity levels (Pfinder et al. 2020; Sassano et al. 2024). The same failure may be repeated for sugar reformulation. It is important to differentiate between reducing sugar in beverages and solid foods. While removing sugar from drinks can provide clear metabolic benefits, replacing sugar in solid foods is more complex and may offer little caloric advantage (Cooper 2017). Unlike sugary drinks, where sugar can often be swapped with non‐caloric sweeteners without major changes to the product, solid foods rely on sugar for more than just sweetness. Replacing sugar in these products often requires several ingredients to replicate its functions, which may not result in lower‐calorie or healthier alternatives (Cooper 2017). Comparing the success of sugar reduction in drinks to that in solid foods oversimplifies the challenge (Goldfein and Slavin 2015).

## 5 Limitations of Modelling Studies

Many arguments supporting sugar reformulation rely heavily on modelling studies that predict large reductions in total energy intake and consequent improvements in obesity rates (Amies‐Cull et al. 2019; Basto‐Abreu et al. 2018; Bernstein et al. 2023). The meta‐analysis by Hashem, He, and MacGregor (2019) reveals that while reformulation can lower sugar intake and offer health benefits, the bulk of the evidence comes from modelling studies based on assumptions, which raises questions about the real‐world applicability of these studies. A significant challenge is the inconsistency in methodologies across studies, including differences in the range of products targeted, which complicates efforts to draw uniform conclusions about the effectiveness of reformulation in reducing sugar and calorie intake (Federici et al. 2019). While the convergence of results across multiple studies with diverse methodologies can enhance confidence in their findings, the nuances of each methodology should still be carefully considered when interpreting outcomes and designing future research.

Experimental evidence from the randomised controlled trials in the meta‐analysis which showed significant and clinically meaningful reductions in sugar intake and bodyweight (Raben et al. 2002; Raben et al. 2011) was short term and was not specifically designed to assess the impact of reformulation itself, but rather focused on substituting regular SSBs with artificially sweetened alternatives. Another two studies (Gatenby et al. 1997; Markey et al. 2016) which looked at replacing full sugar products with reduced sugar products found that while sugar intake was reduced, it did not result in clinically meaningful changes in bodyweight. Observational and modelling studies suggest some health improvements, but the effect was generally small and the overall quality of the evidence was graded as low to very low (Hashem, He, and MacGregor 2019), largely due to design flaws and reliance on self‐reported dietary data, which are prone to underreporting (Ottaviani et al. 2024).

An additional concern is that many modelling studies assume consumer behaviour will remain unchanged after reformulation, which is unlikely (Amies‐Cull et al. 2019). For example, a widely cited modelling study by Ma et al. (2016) predicting that a 40% reduction in free sugars could prevent 1.5 million cases of overweight and obesity in the United Kingdom failed to account for compensatory behaviours like larger portions or switching to other high‐calorie foods, making the estimates less reliable. Moreover, modelling studies often overlook the technical challenges and nutritional trade‐offs in reformulating solid foods, which can limit the overall impact on calorie intake. When these factors are considered, the effect of reformulation on population calorie reduction is often minimal, as was seen with the United Kingdom's Sugar Reduction Programme, even when 25% of added sugars were removed from all packaged foods (Yeung et al. 2017).

These limitations underline the need for more robust, long‐term studies that take into account real‐world consumer behaviour and the complexities of reformulation. While modelling studies can offer valuable insights, they should not be the sole basis for shaping sugar reformulation policies.

## 6 Unintended Consequences and Limitations of the Sugar‐Centric Approach

Beyond these technical challenges, sugar reduction in solid foods may lead to several unintended consequences that could undermine its effectiveness as an obesity prevention strategy. One significant concern is the ‘health halo’ effect (Cao et al. 2022). Products labelled as ‘reduced sugar’ or ‘low sugar’ may be perceived as healthier by consumers, regardless of their nutritional profile (Prada et al. 2021). This perception can lead to overconsumption, as individuals may feel less guilty about eating larger portions or consuming these products more frequently.

The singular focus on sugar reduction may lead to neglecting other important nutritional considerations, potentially resulting in products that are lower in sugar but less nutritionally balanced overall (Cooper 2017). Erickson and Slavin (2015b) found that following restrictive added sugar guidelines could lead to nutritionally inadequate diets in other areas, such as fibre and certain micronutrients. Moreover, the push for sugar reduction may increase the use of artificial sweeteners, which remain controversial regarding their long‐term health effects and potential impact on appetite regulation (World Health Organization 2023). A large‐scale prospective study by Fowler et al. (2008) found that regular consumption of artificially sweetened beverages was associated with increased long‐term weight gain, contrary to their intended effect.

Overemphasis on sugar may divert attention and resources from other crucial elements that contribute to the obesity epidemic, such as portion sizes, food environment, physical activity and socioeconomic factors. A narrow focus on sugar reduction risks demonising a specific nutrient, potentially leading to unhealthy dietary restrictions or anxiety around food choices (Russell et al. 2022). The current approach to sugar reduction bears striking similarities to the previous mistake made by the public health movement regarding dietary fat in combating obesity (Ebbeling et al. 2018; Harcombe et al. 2015; La Berge 2008). In the late 20th century, the vilification of dietary fat led to a proliferation of low‐fat products that were often high in sugar and refined carbohydrates (Ebbeling et al. 2018). This shift not only failed to curb obesity rates but may have contributed to their increase (La Berge 2008). By focusing too narrowly on sugar as the primary culprit in obesity, we risk oversimplifying a complex issue and potentially driving consumers towards other unhealthy dietary choices.

The creation of a plethora of ‘lower’ sugar, nutrient‐poor products with many food additives to replace the function of sugar raises questions about whether this approach truly benefits public health. These reformulated products remain energy‐dense and nutrient‐poor and may not offer significant nutritional advantages over their original counterparts. We must question the value of ‘lower sugar’ reformulations, such as double chocolate chip cookies that use additives to replace the functions of sugar to maintain organoleptic properties, minimally reduced total calories and often increase fat content. Is this truly healthier than the original? No. These products create an illusion of health without significant nutritional improvement.

While consumer education has been a prominent strategy for the past three decades, its implementation has achieved limited success in changing dietary behaviours at a population level. Despite this, dietary guidelines continue to allow for some consumption of energy‐dense, nutrient‐poor products. However, meeting these guidelines would require dramatic reductions in current consumption patterns—for instance, UK dietary guidelines would necessitate more than a 50% decrease in the intake of foods high in fat and sugar (Scarborough et al. 2016). Although complete elimination of these foods from the diet is often unrealistic and may lead to feelings of deprivation or binge eating (Horovitz and Argyrides 2023), simply advocating for ‘moderation’ without concrete guidance has proven insufficient. Future educational initiatives would need to be substantially reformed to be effective, potentially incorporating elements such as practical skills training in meal planning, clear quantitative guidelines for portion sizes and frequency of consumption, and addressing the environmental and social factors that influence food choices. This more comprehensive approach, combined with the principles of intuitive eating (Van Dyke and Drinkwater 2014), could help individuals develop a more informed and balanced relationship with food while working towards the substantial dietary changes required for better health outcomes.

## 7 A Way Forward: A Comprehensive Approach to Obesity Prevention

Given the complexities and potential limitations of sugar reformulation in solid foods, a more holistic approach to obesity prevention that addresses multiple factors without creating undue focus on any single nutrient should be adopted. This approach aligns with emerging research suggesting that overall dietary patterns are more important for health outcomes than manipulating individual nutrients (Martini et al. 2023). The Mediterranean diet, for example, has been associated with numerous health benefits, including weight management, despite not specifically restricting sugar intake (D'Innocenzo et al. 2019).

Supporting and strengthening existing food‐based dietary guidelines that emphasise overall dietary patterns remains vital. While these guidelines already promote a holistic view of nutrition that goes beyond single nutrients (Cámara et al. 2021; Herforth et al. 2019), implementation strategies need to better help people understand and apply pattern‐based approaches in their daily food choices (Gabe, Tramontt, and Jaime 2021). This includes considering how different foods work together as part of healthy eating patterns, the cultural and social contexts of meals and the practical aspects of planning and preparing balanced diets that align with recommended dietary patterns.

Equally important is improving the food environment through policies that enhance access to affordable, nutritious foods, especially in underserved areas (Ziso, Chun, and Puglisi 2022). Promoting physical activity is another key element in preventing obesity. Additionally, supporting evidence‐based behavioural interventions that address the psychological and social drivers of eating habits is crucial to fostering long‐term healthy behaviours.

Rather than a blanket approach to sugar reduction, reformulation efforts should focus on products where such changes are likely to have the most significant impact without compromising overall nutritional quality or leading to unintended consequences. For example, reducing sugar in SSBs could be more beneficial than reformulating nutrient‐dense foods where sugar plays multiple functional roles. The evidence clearly shows that people consistently consume more food and drink when offered larger portions, packages or tableware (Hollands et al. 2015). While emphasising appropriate portion sizes across all food categories remains important, we need to better understand the complex relationship between portion‐size reduction and consumption behaviour (Rolls 2014). Critical research gaps exist around identifying the threshold at which reducing portion sizes may lead to compensatory behaviours, such as consuming multiple units instead of one (Haynes et al. 2019). This ‘tipping point’ likely varies across different food categories, eating occasions and consumer groups (Vermeer, Steenhuis, and Poelman 2014). Understanding these nuances through targeted research could help design more effective portion‐size interventions that achieve genuine reductions in overall consumption rather than inadvertently encouraging multiple‐unit consumption. Additionally, research should examine how portion‐size strategies interact with other factors like pricing, packaging design and point‐of‐sale messaging to influence consumer behaviour (Chu, Tang, and Hetherington 2024; Vermeer, Steenhuis, and Poelman 2014).

Investing in longitudinal research to assess the impact of various obesity prevention strategies, including sugar reformulation, on population health outcomes is essential. Such studies can provide valuable insights into different interventions' long‐term effects and potential unintended consequences. Simultaneously, addressing the social determinants of health that contribute to obesity, including poverty, food insecurity and lack of access to healthcare, is crucial for comprehensive obesity prevention (Kumanyika 2019). Fostering collaboration with the food industry to develop innovative solutions that improve the overall nutritional quality of processed foods without compromising taste, affordability or safety is essential (Capozzi et al. 2021; Weaver et al. 2014). While this may seem at odds with critiquing sugar reformulation, the key is to focus on holistic improvements rather than single‐nutrient reductions.

This comprehensive approach recognises the complexity of the obesity epidemic and avoids the pitfalls of oversimplified solutions that may ultimately do more harm than good. It aligns with the growing evidence suggesting that multifaceted interventions are more effective in addressing complex public health issues like obesity (Bagnall et al. 2019). They demonstrate that effective obesity prevention requires a systems‐level approach considering the complex interplay of individual behaviours, social norms and environmental factors (Bagnall et al. 2019).

## 8 Concluding Remarks

The global obesity epidemic presents a complex public health challenge that requires balanced, multifaceted solutions. While reducing sugar content in foods has been proposed as a potential intervention, its efficacy is subject to debate, particularly when applied to solid foods. This article challenges the mainstream belief in the universal benefits of sugar reformulation and highlights the need for a more comprehensive approach to obesity prevention.

Evidence suggests that the impact of sugars from SSBs differs from that of solid foods, complicating the application of a uniform sugar reduction strategy. Technical challenges in reformulating solid foods, limitations of modelling studies and potential unintended consequences further question the efficacy of sugar reformulation as a primary obesity prevention strategy. While sugar reduction, particularly in SSBs, may play a role in addressing health concerns, the complexity of obesity necessitates a multifaceted approach that addresses various contributing factors and promotes overall dietary quality. It is crucial to recognise that simplistic, single‐nutrient‐focused interventions may overlook other important factors contributing to obesity.

While the intention behind sugar reformulation is commendable, its application, particularly to solid foods, may not yield the desired results in obesity prevention. Moving beyond simplistic interventions allows for developing more robust and sustainable solutions to this complex public health challenge.

## Conflicts of Interest

The author declares no conflicts of interest.

## Declaration of Generative AI and AI‐Assisted Technologies in the Writing Process

During the preparation of this work, the author used Claude 3.5 sonnet in order to improve the grammar and clarity of the text they drafted. After using this tool/service, the corresponding author reviewed and edited the content as needed and takes full responsibility for the content of the publication.

## Data Availability

Data sharing not applicable‐no new data generated, or the article describes entirely theoretical research.
